# Supplementary material for: Causal Relationship between Aging and Anorexia Nervosa: A White-Matter-Microstructure-Mediated Mendelian Randomization Analysis
Source: Biomedicines. 2024 Aug 16;12(8):1874. doi: 10.3390/biomedicines12081874 (PMC11351342; doi:10.3390/biomedicines12081874)
Supplement: Supplementary file 1 [file biomedicines-12-01874-s001.zip › Supplementary Figure/Figure S8/Leaveoneout plot of MR analysis for TL to ubm-b-1911.pdf]

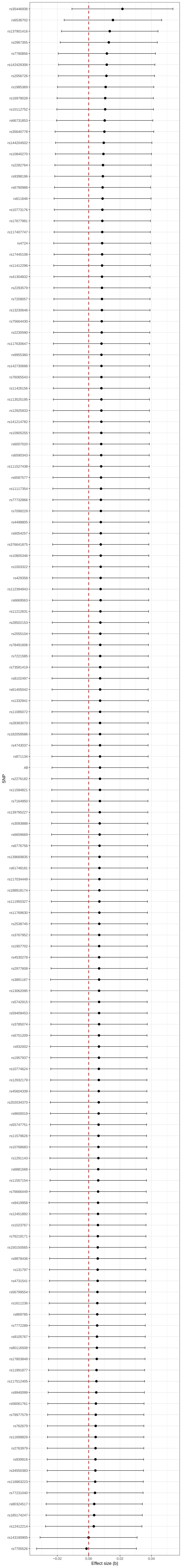
SNP
Effect size (b)
-0.02
0.00
0.02
0.04
rs35446936
rs6536702
rs137901416
rs2967355
rs7790856
rs142426306
rs2056726
rs1985369
rs16978028
rs10112752
rs66731853
rs35640778
rs144204502
rs10840270
rs2282764
rs9398196
rs6790988
rs611646
rs10773176
rs17677991
rs117407747
rs4724
rs17445108
rs11422296
rs41304832
rs2293579
rs7209057
rs13230646
rs75664430
rs2230590
rs117630647
rs9955360
rs142730696
rs76065543
rs11426156
rs113525195
rs12925933
rs141214782
rs10905255
rs6007020
rs6590343
rs111527438
rs6587577
rs11117354
rs77732866
rs7099229
rs4498805
rs6054257
rs376641875
rs10805346
rs1003322
rs429358
rs112394943
rs6669563
rs11212631
rs28502153
rs2555104
rs78491606
rs7221585
rs73581419
rs8102497
rs61405042
rs1332941
rs11085072
rs28363070
rs182059586
rs4743037
rs871134
All
rs2276182
rs11584821
rs7164950
rs139795227
rs3093888
rs6659669
rs6776756
rs139669835
rs61748181
rs117034449
rs188918174
rs111950327
rs11769630
rs2538745
rs3767952
rs1907702
rs4530278
rs2977608
rs3891167
rs13062095
rs5742915
rs59409453
rs3785074
rs6751209
rs932002
rs1957937
rs10774624
rs12932179
rs45604339
rs202034370
rs9600019
rs55747751
rs11579626
rs10768683
rs1291143
rs6881568
rs11557154
rs76666449
rs9419958
rs12451892
rs1023767
rs76219171
rs150150565
rs9878436
rs131797
rs4731541
rs56799554
rs1611236
rs869785
rs7772289
rs8105767
rs80116508
rs17803849
rs11991877
rs117512405
rs9940099
rs56061761
rs79977579
rs762679
rs11699829
rs2763979
rs939916
rs34550383
rs116863223
rs77231040
rs80324517
rs185174247
rs12412214
rs143190905
rs7705526
